# Clinical Utility of Baseline Brain Natriuretic Peptide Levels on Health Status Outcomes after Catheter Ablation for Atrial Fibrillation in Individuals without Heart Failure

**DOI:** 10.3390/jcm13020407

**Published:** 2024-01-11

**Authors:** Shin Kashimura, Nobuhiro Ikemura, Shun Kohsaka, Yoshinori Katsumata, Takehiro Kimura, Daisuke Shinmura, Kotaro Fukumoto, Koji Negishi, Ikuko Ueda, Seiji Takatsuki, Masaki Ieda

**Affiliations:** 1 Department of Cardiology, Yokohama Municipal Citizen’s Hospital, 1-1, Mitsuzawa-nishicho, Kanagawa-ku, Yokohama 221-0855, Japanshiminkn@gmail.com (K.N.); 2 Department of Cardiology, Keio University School of Medicine, 35 Shinanomachi, Shinjuku, Tokyo 160-8582, Japangoodcentury21@keio.jp (Y.K.); veritas@bp.iij4u.or.jp (T.K.); iueda@a7.keio.jp (I.U.); mieda@z8.keio.jp (M.I.)

**Keywords:** atrial fibrillation, brain natriuretic peptide, catheter ablation, non-heart failure, quality of life

## Abstract

Background: Catheter ablation (CA) benefits atrial fibrillation (AF) patients with heart failure (HF). Brain natriuretic peptide (BNP), a marker of left-ventricular pressure load, may serve as a potential surrogate for predicting quality of life (QOL) in a broader range of patients. Methods: Within the multicenter KiCS-AF registry, 491 AF patients underwent CA without clinical HF (e.g., documented history of HF, left ventricular ejection fraction ≤ 40%, or BNP levels ≥ 100 pg/mL). Participants, aged 61 ± 10 years, were categorized by baseline BNP quartiles. Atrial Fibrillation Effect on QualiTy-of-Life (AFEQT) questionnaire assessments were assessed at baseline and 1 year. Results: A lower baseline BNP correlated with reduced AFEQT scores. Post CA, all groups showed significant AFEQT score improvements. The lower-BNP group displayed notable enhancements (18.2 ± 1.2, 15.0 ± 1.1, 12.6 ± 1.2, 13.6 ± 1.2, *p* < 0.005), especially in symptom and treatment concern areas. Even those with normal BNP levels (≤18.4 pg/mL) exhibited significant QOL improvements. Comparing paroxysmal AF (PAF) and non-PAF groups, the PAF group, especially with higher BNP levels, showed greater AFEQT score improvements. Conclusions: This study establishes BNP as a predictive marker for QOL enhancement in non-HF patients undergoing CA for AF. BNP levels represent AF stages, with individuals in earlier stages, especially within normal BNP levels, experiencing greater QOL improvements.

## 1. Introduction

Atrial fibrillation (AF) is one of the most prevalent forms of atrial arrhythmias among adult patients, with a rising incidence due to an aging society. Age, in tandem with heart failure (HF), is linked to the emergence of AF, and vice versa. The concurrent presence of both conditions significantly amplifies mortality risks [1]. The Framingham Heart Study found that 37% of patients with newly diagnosed AF had HF and 57% of patients with newly diagnosed HF had AF [2]. These findings underscore the importance of identifying HF or its risk factors in patients with AF. In this regard, plasma brain natriuretic peptide (BNP) emerges as a well-established indicator of congestive HF, capable of detecting HF in its early stages. Elevated BNP levels in patients pose a risk of HF development, irrespective of their underlying heart disease and the left ventricular ejection fraction (LVEF) value [3].

BNP is frequently used as a surrogate marker to discern subtle changes in LV function arising from AF, making it a crucial indicator in assessing responses to specific AF treatments, such as catheter ablation (CA). While CA for AF improves patients’ quality of life (QOL), the clinical utility of BNP levels on the QOL benefit after CA remains uncertain. Therefore, in this study, we aimed to investigate the association of baseline plasma BNP levels and QOL changes after undergoing CA in AF patients without a history of clinical HF.

## 2. Methods

### 2.1. Data Source

We obtained patient data from the Keio interhospital Cardiovascular Studies-atrial fibrillation (KiCS-AF) registry. In brief, the KiCS-AF registry [4] is a prospective, multicenter, registry-based cohort study designed to collect data on the clinical variables and outcomes of consecutive patients with AF from 11 hospitals within the Tokyo metropolitan area of Japan from September 2012 to May 2018 (UMIN 000022229). The Atrial Fibrillation Effect on Quality-of-Life (AFEQT) questionnaires [5] were administered to all patients at the baseline visit and at the 1-year follow-up visit or by mail, if possible. Yearly follow-up examinations were performed for all patients through chart reviews, mail, and phone interviews. Data quality assurance was achieved through systematic validation that highlighted outliers and data completeness, and the clinical research coordinators in each institution answered all inquiries regarding data entry [4]. To ensure consecutive case enrollment, the senior study coordinator (I.U.) and investigator (S.Ko.) performed on-site auditing to ensure proper registration of each eligible patient. The registry was conducted per the tenets of the Declaration of Helsinki. Institutional review board/ethics committee approval (Ethic Committee Name: Keio University School of Medicine Ethics Committee, Approval Code: 20120029, Approval Date: 20 April 2012) was obtained from all the study sites. All patients provided written informed consent [4].

### 2.2. Studied Patients

**Figure 1:**
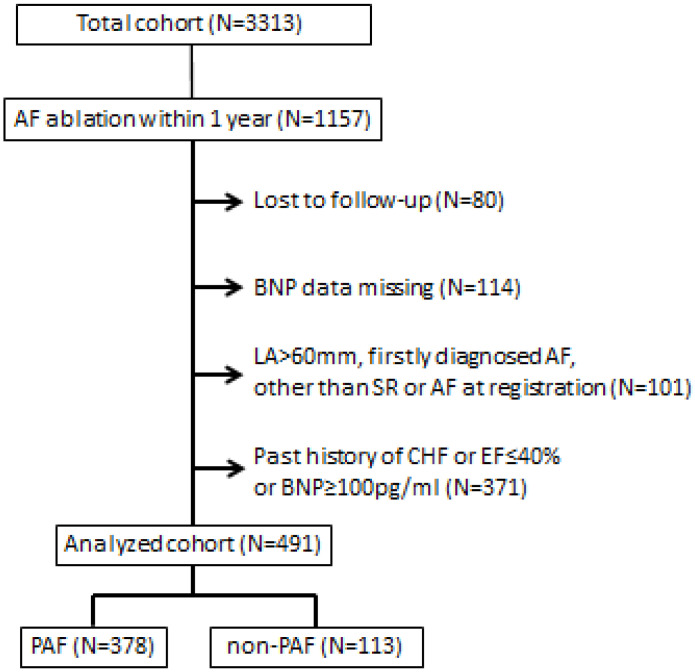
Flowchart of the patients analyzed.

The study flowchart is illustrated in Figure 1. A total of 3313 consecutive patients with AF were registered in this study. Among those, 1157 patients underwent CA for AF within 1 year after registration, and the 1-year follow-up data of 1077 patients were available. Of those, data regarding plasma BNP levels were available in 963 patients. The exclusion criteria were left atrial (LA) diameter ≥ 60 mm by transthoracic echocardiogram, or a 12-lead electrocardiogram at registration showing a rhythm other than the sinus rhythm (SR) or AF. A first episode of AF was also excluded. The recurrence rate of these patients was only 50% in 1 year after diagnosis, despite CA being performed only for less than 3% of them [6]. Finally, we analyzed 491 non-HF patients (mean age, 61 ± 10 years; 385 (78%) males; and 378 (77%) patients with paroxysmal AF (PAF)). HF was defined as having a history of HF, an LVEF of ≤40%, or a plasma BNP level of ≥100 pg/mL [7]. Enrolled patients were divided into quartiles based on their plasma BNP levels (normal, ≤18.4 pg/mL [8]) before undergoing CA as follows: Q1: 1.32–18.5 pg/mL, Q2: 18.7–37.7 pg/mL, Q3: 37.9–63.5 pg/mL, and Q4: 63.8–99.8 pg/mL.

In this study, the primary aim of performing CA was to ensure that all pulmonary veins are electrically isolated and SR is restored. Performing an additional ablation procedure and prescribing postoperative antiarrhythmic drugs (AADs) depended on the operator. After ablation, all patients received anticoagulation therapy for at least 6 months. Anticoagulation rates 1 year after CA were comparable among quartiles.

### 2.3. Assessment of Patients’ Health Status

We evaluated the patient-reported health status at the time of registration (before CA) and 1 year after registration (after CA) using the AFEQT questionnaire [5]. The AFEQT consists of four conceptual domains (symptoms, daily activities, treatment concern, and satisfaction), and its global score was used to measure the patient’s AF-specific health status in this study. This score ranges from 0 to 100, where 100 indicates the best AF-related health status and 0 indicates the worst. A culturally and linguistically translated version of the AFEQT for Japan was used.

### 2.4. Statistical Analysis

Continuous variables are presented as the mean ± standard deviation, and categorical variables are presented as numbers and percentages. Comparisons among the quartiles were performed using the one-way analysis of variance for continuous variables and the Kruskal–Wallis test for ordinal variables. Differences between the groups were compared using the Student’s *t*-test or Mann–Whitney U test. Changes in the AFEQT scores before and after CA were analyzed by multivariable linear regression with generalized estimating equations to account for relevant factors, including the baseline AFEQT score (per 1-point increase), age (per 1-year increase), PAF presence, beta blocker use, and estimated glomerular filtration rate (eGFR). In this study, all 491 patients analyzed had effective BNP data. A *p*-value of <0.05 was considered significant. These analyses were performed using EZR software v1.36 (Saitama Medical Center, Jichi Medical University, Saitama, Japan).

## 3. Results

### 3.1. Baseline Characteristics

Table 1 presents the baseline characteristics of non-HF groups stratified by quartiles based on BNP levels. Patients with higher BNP levels were found to be older, with ages ranging from 57 ± 10 years in Q1 to 64 ± 10 years in Q4 (*p* < 0.001). This age difference correlated with a higher CHA2DS2-Vasc score, increasing from 1.1 ± 1.1 in Q1 to 1.7 ± 1.3 in Q4 (*p* < 0.005). The higher BNP groups also exhibited a lower prevalence of PAF, with percentages decreasing from 96% in Q1 to 57% in Q4 (*p* < 0.001). Furthermore, patients in the higher BNP quartiles were more frequently prescribed beta blockers, with percentages rising from 33% in Q1 to 53% in Q4 (*p* < 0.01). Conversely, the distribution of patients with hypertension, diabetes mellitus, a history of cerebral infarction or transient ischemic attack, and the prescription of AADs, along with the percentage of male patients, remained comparable across the quartiles.

As for the echocardiographic variables, the higher-BNP groups exhibited noteworthy differences. The LA diameter increased from 3.7 ± 0.6 in Q1 to 4.0 ± 0.7 in Q4 (*p* < 0.001) among the higher BNP quartiles. Concomitantly, the LA appendage flow velocity showed a decrease from 67 ± 21 in Q1 to 52 ± 21 in Q4 (*p* < 0.001) within the same quartile comparison.

### 3.2. Improvement in AFEQT Score

Prior to undergoing CA, the lower-BNP group exhibited lower overall AFEQT scores in both the symptom and treatment concern domains, as indicated in Table 1. However, following CA, AFEQT scores became comparable across all domains, as shown in Table 2 (upper row). Notably, in comparison to the scores pre-CA, all quartiles demonstrated a significant increase in the total AFEQT score within each domain.

**Figure 2:**
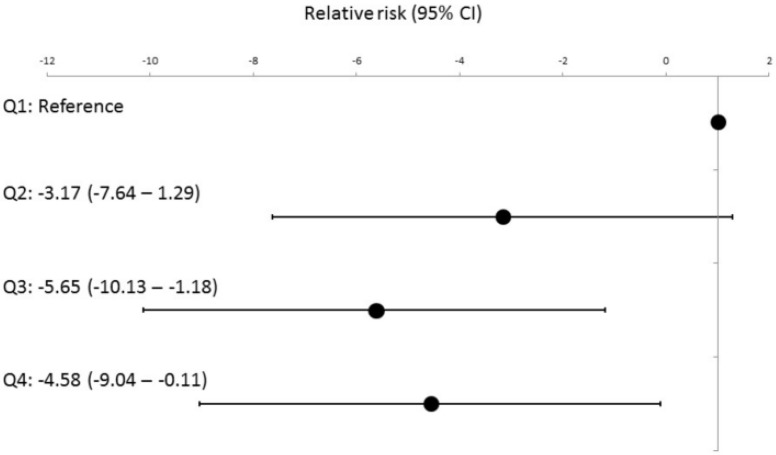
Impact of plasma brain natriuretic peptide levels on changes in the AFEQT score after catheter ablation in patients with atrial fibrillation. Q1: 1.32–18.5 pg/mL, Q2:18.7–37.7 pg/mL, Q3: 37.9–63.5 pg/mL, and Q4: 63.8–99.8 pg/mL.

Moreover, the lower BNP quartile displayed a noteworthy enhancement in the overall AFEQT score, particularly in the symptom and treatment concern domains, after undergoing CA (Table 2 (lower row)). After adjusting for the clinically relevant factors including baseline AFEQT score (Supplemental Table S1) and confounding factors such as age, beta blocker use, eGFR, and PAF (Supplemental Table S2), lower baseline BNP level scores were significantly associated with a greater AFEQT score improvement at 1 year (Figure 2), especially for the symptom and treatment concern domains (Supplemental Figure S1).

Furthermore, a comparison between normal and abnormal BNP groups revealed that the normal BNP (≤18.4 pg/mL) group experienced a significant improvement in QOL, evident in the overall AFEQT score and the symptom and treatment concern domains (Table 3).

### 3.3. Comparative Analysis of PAF and Non-PAF Groups

**Figure 3:**
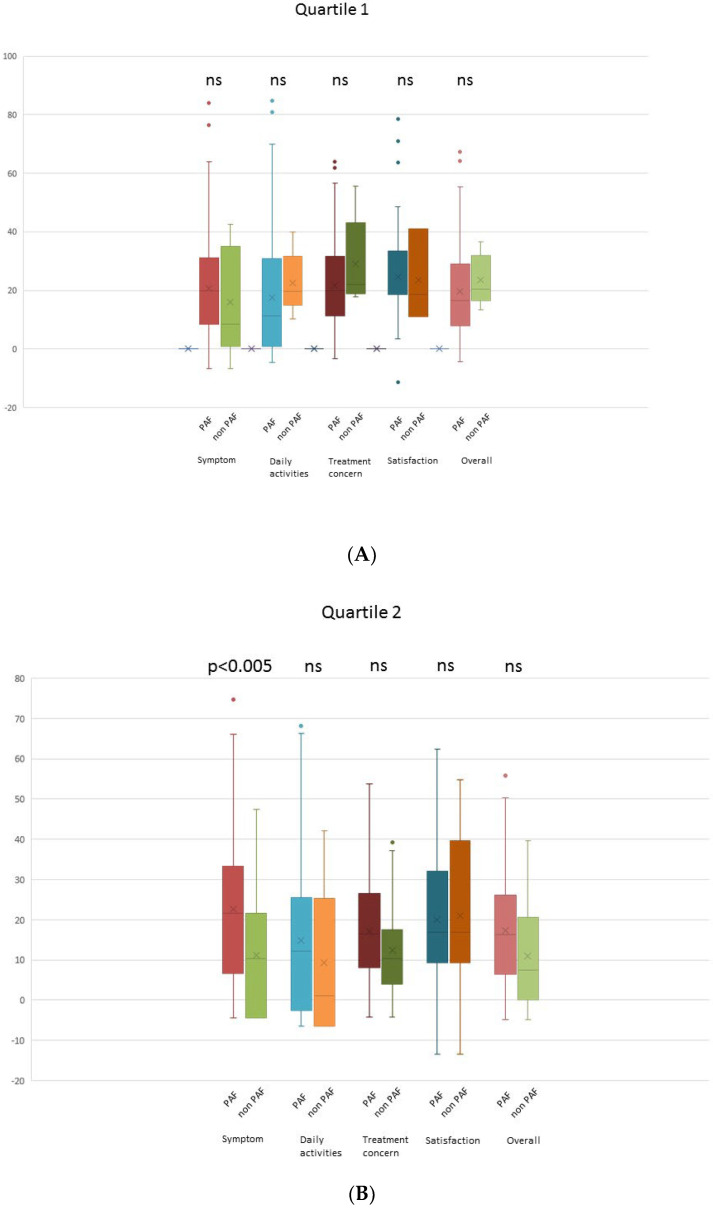

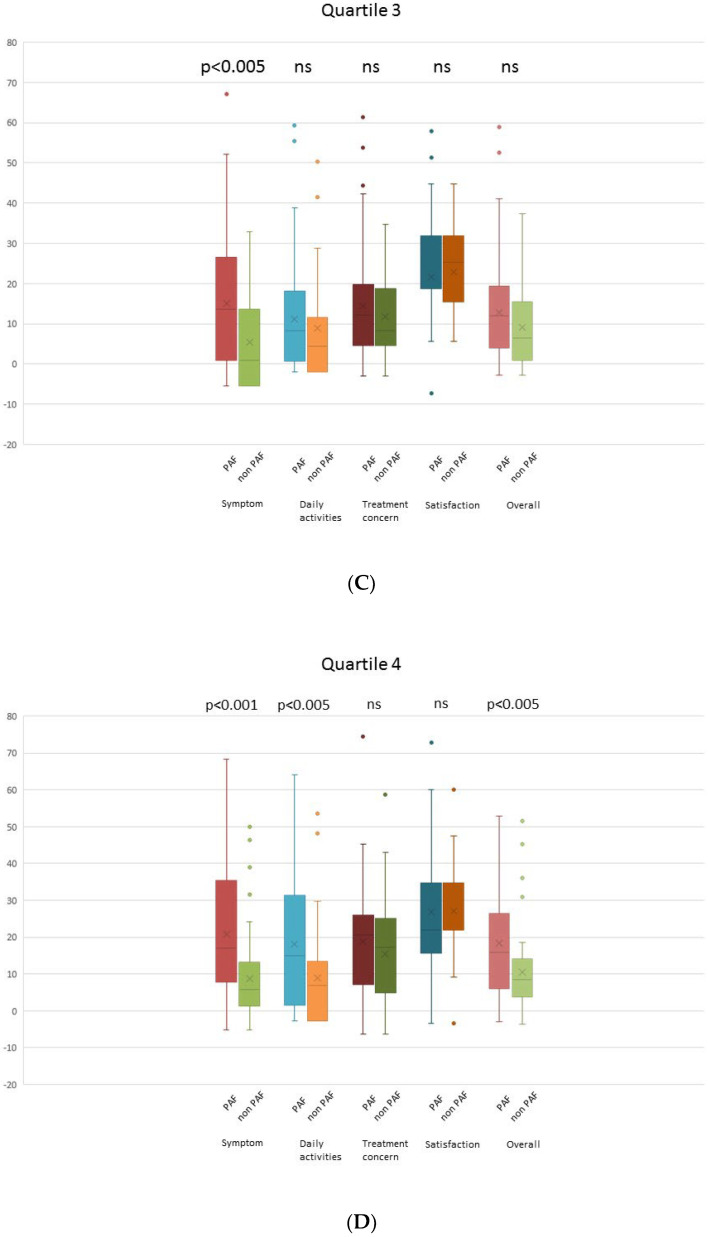
Comparison of changes in the AFEQT scores after catheter ablation between patients with PAF and those with non-PAF. (**A**) Quartile 1, (**B**) quartile 2, (**C**) quartile 3, and (**D**) quartile 4. Q1: 1.32–18.5 pg/mL, Q2: 18.7–37.7 pg/mL, Q3: 37.9–63.5 pg/mL, and Q4: 63.8–99.8 pg/mL.

When comparing the PAF and non-PAF groups, it was observed that changes in the AFEQT score across all domains were not significantly different in quartile 1 (lowest BNP group). However, in the other quartiles (higher BNP groups), a noteworthy finding emerged: the PAF group demonstrated a significantly greater improvement in the AFEQT score, particularly in the symptom domain. Importantly, this trend persisted even after adjusting for the baseline AFEQT score. Intriguingly, patients with PAF in the highest BNP group exhibited a significantly greater improvement in both the overall AFEQT score and the daily activities domain, as illustrated in Figure 3.

## 4. Discussion

This study represents the inaugural investigation to reveal a positive correlation between lower baseline plasma BNP levels and an enhanced QOL in non-HF patients who underwent CA for AF. Specifically, those with lower plasma BNP levels achieved higher scores in the symptom and treatment concern domains of the AFEQT.

While the effectiveness of CA for AF in patients with HF has been extensively explored, limited knowledge exists regarding the extent of QOL improvement and the influence of plasma BNP levels before CA in patients without HF. This study contributes novel insights to the interplay between plasma BNP levels and QOL after CA in non-HF patients with AF, shedding light on a previously unexplored aspect of this therapeutic intervention. Previously, the CASTLE-AF trial showed that performing CA for AF in patients with HF reduced the mortality from any cause and hospitalization for worsening HF [9]. The disappearance of an atrial kick is widely known to cause reduced cardiac function. Furthermore, the target heart rate in patients with AF [10], the irregularity of cardiac rhythm during AF [11], and the neuroendocrine function of the atrium [12] have been recognized as key determinant factors for adverse effects in patients with HF. The restoration of SR through CA in patients with congestive HF significantly improved their QOL [13]. The CAMTAF trial also showed an improvement in the LVEF and HF symptoms among patients with persistent AF after CA [14]. A systematic review [15] showed that performing CA for AF significantly improved the patient’s QOL both physically and mentally, and patients without recurrence had a greater improvement in their QOL than those who experienced recurrence. Even among patients with asymptomatic AF, the maintenance of SR by CA was reported to improve their QOL, exercise performance, and plasma BNP levels [16].

Among patients with non-HF, elevated N-terminal prohormone BNP levels were reported to be correlated with stroke, all-cause death, and hospitalization for HF [17]. An increased plasma BNP level is known to correlate not only with an increased incidence of AF [18] but also with an elevated mortality rate [19]. Furthermore, the BNP level decreases after successful CA for AF and correlates with the atrial arrhythmia burden after AF ablation [20].

In our current investigation, recognizing that plasma BNP levels tend to rise in patients with AF, patients demonstrating normal BNP levels and presenting with PAF were considered to possess unimpaired cardiac function, coupled with a relatively infrequent occurrence of AF episodes, notably without recent manifestations. This subgroup of patients demonstrated a favorable response to CA for AF, resulting in a notable improvement in their AFEQT score. The analysis of individual domains of the AFEQT questionnaire, particularly the symptom domain, revealed that lower BNP levels were indicative of a reduced AF burden. This suggests that patients with lower BNP levels with a lower AF burden are more sensitive to AF, and CA for AF will improve their QOL more greatly. Verma et al. reported that the ratio of asymptomatic to symptomatic AF had increased after CA [21]. Previous studies have stated that younger patients [22] and those with PAF [23] tended to be more symptomatic to AF. Similarly, our study data showed that patients with lower BNP levels, including younger individuals and those with PAF, exhibited greater symptomatic responses to AF.

Regarding the treatment concern domain, we noted only a marginal increase in plasma BNP levels shortly after the onset of AF. It is conceivable that these patients may have experienced anxiety, fearing the onset of AF at any moment or as a potential side-effect of treatment. However, the degree of QOL improvement in the daily activities and satisfaction domains was comparable among the quartiles. Yanagisawa et al. [24] reported that CA improved the number of maximum daily steps performed in parallel in patients with PAF and those with non-PAF. The dissatisfaction among patients with CA for AF has been attributed not only to procedural failure but also to excessive pain experienced during the procedure [25].

Concerning the type of AF, CA for PAF in patients without HF resulted in a greater improvement in QOL than in those with non-PAF among the higher-BNP groups. This enhancement in QOL after CA was similar between patients with PAF and those with non-PAF in the lowest-BNP group. These individuals were considered to be in the early stages of AF, and their AF burden was comparable. Generally, PAF is more susceptible to treatment than non-PAF using CA. In this study, as higher BNP quartiles included a greater number of patients with non-PAF, the magnitude of QOL improvement among the patients in lower BNP quartiles was greater than that among those in higher BNP quartiles. This outcome may reflect the proportion of patients with PAF and the success of SR restoration after CA.

Charitakis et al. [26] reported that anxiety and LA dilatation predicted the occurrence of arrhythmia-related symptoms. However, the present study yielded conflicting results as patients in the lower BNP groups, characterized by a smaller LA diameter, exhibited lower AFEQT scores in both the treatment concern (reflecting the severity of anxiety) and symptom domains. This discrepancy may be attributed to the higher proportion of patients with PAF enrolled in our study (77% vs. 37%). Consequently, individuals with a smaller LA diameter in this group may not have become accustomed to AF and, as a result, experienced a lower QOL due to AF attacks rather than HF symptoms caused by atrial enlargement.

This study included patients with both PAF and non-PAF, suggesting potential benefits from CA in terms of QOL. The presence of normal BNP levels, indicative of an earlier stage of AF regardless of its type, suggests an optimal opportunity for achieving the best response to CA. Furthermore, performing CA for AF in its early stage is believed to arrest the progression of the condition before developing HF.

## 5. Limitations

This study is subject to certain limitations. The questionnaire of AFEQT was translated into Japanese, it does not necessarily reflect the completely same psychometric properties as the original paper. Unmeasured confounding factors, such as the patient’s psychiatric status or frailty, or other pertinent clinical factors, may have influenced the results. The primary focus of this study was on the improvement in QOL through CA for AF. Additionally, the relationship between the actual percentage of SR restoration and QOL improvement remains unclear. To address this unresolved question, the consideration of an implantable loop recorder may provide valuable insights.

## 6. Conclusions

This study demonstrates that individuals with non-HF and AF exhibiting lower BNP levels experience a more substantial improvement in QOL following CA compared to those with higher BNP levels. Moreover, patients in the lowest BNP group exhibited an enhanced QOL after CA, regardless of the type of AF. Therefore, considering the improvement in QOL, CA should be regarded as a viable option for individuals in the early stages of AF, particularly those with normal BNP levels.

## Figures and Tables

**Table 1:** Baseline characteristics.

Characteristics, Count (%)	Quartile 1 (Lowest, 1.32–18.5) *n* = 123	Quartile 2 (18.7–37.7) *n* = 123	Quartile 3 (37.9–63.5) *n* = 122	Quartile 4 (Highest, 63.8–99.8) *n* = 123	*p* Value
Age, years, mean ± SD	57 ± 10	61 ± 10	62 ± 11	64 ± 10	<0.001
Female	19 (15)	26 (21)	31 (25)	30 (24)	0.224
BMI, kg/m^2^	24 ± 3	24 ± 3	24 ± 3	24 ± 4	0.706
Hear rate, bpm	74 ± 14	73 ± 16	73 ± 16	78 ± 17	0.05
Comorbidities					
Hypertension	55 (48)	53 (43)	58 (48)	61 (50)	0.743
Diabetes Mellitus	10 (8)	16 (13)	13 (11)	14 (11)	0.665
Dyslipidemia	46 (37)	40 (33)	40 (33)	45 (37)	0.793
Stroke or TIA	7 (6)	7 (6)	4 (3)	7 (6)	0.777
Obstructive sleep apnea	7 (6)	6 (5)	6 (5)	2 (2)	0.401
Coronary artery disease	2 (2)	3 (2)	8 (7)	2 (2)	0.075
Sick sinus syndrome	4 (3)	3 (2)	7 (6)	4 (3)	0.547
eGFR, mL/min/1.73 m^2^	71 ± 13	68 ± 14	67 ± 13	65 ± 12	<0.01
CHADS_2_ score	0.7 ± 0.8	0.7 ± 0.9	0.7 ± 0.8	0.9 ± 0.9	0.408
CHA_2_DS_2_-Vasc score	1.1 ± 1.1	1.3 ± 1.2	1.5 ± 1.3	1.7 ± 1.3	<0.005
Paroxysmal AF	118 (96)	102 (83)	89 (73)	69 (57)	<0.001
Medications					
Beta blocker	40 (33)	61 (50)	50 (41)	65 (53)	<0.01
Antiarrhythmics	53 (43)	54 (44)	37 (30)	43 (35)	0.083
Echocardiography					
LVEF, %	60 ± 5	59 ± 3	59 ± 4	59 ± 5	<0.05
Left atrium diameter, cm	3.7 ± 0.6	3.8 ± 0.6	4.0 ± 0.7	4.0 ± 0.7	<0.001
E/e’	7.5 ± 2.4	9.5 ± 5.7	8.5 ± 3.5	8.7 ± 3.3	<0.005
LAAFV, cm/sec	67 ± 21	61 ± 19	57 ± 21	52 ± 21	<0.001
AFEQT scores					
Overall	71.1 ± 18.4	74.7 ± 17.0	77.7 ± 17.6	75.4 ± 18.6	<0.05
Symptom	69.7 ± 21.6	71.8 ± 21.1	76.7 ± 20.3	76.7 ± 19.1	<0.05
Daily Activities	75.0 ± 22.8	77.4 ± 21.5	79.7 ± 21.1	76.2 ± 24.3	0.403
Treatment concern	66.4 ± 19.3	72.7 ± 17.4	75.5 ± 18.3	73.2 ± 17.7	<0.001
Satisfaction	59.8 ± 20.8	63.2 ± 19.9	62.7 ± 18.3	60.3 ± 22.0	0.559

All data are expressed as the mean ± standard deviation or *n* (%). Abbreviations, BMI = body mass index; TIA = transient ischemic attack, eGFR = estimated glomerular filtration rate; LAAFV = left atrial appendage flow velocity; LVEF = left ventricular ejection fraction; AFEQT = Atrial Fibrillation Effect on Quality-of-Life.

**Table 2:** The AFEQT score at 1 year follow-up and the absolute changes in AFEQT score.

	Quartile 1	Quartile 2	Quartile 3	Quartile 4	*p* Value
AFEQT scores at 1 year follow-up					
Overall	90.8 ± 10.3	90.8 ± 10.4	89.6 ± 12.4	90.2 ± 11.3	0.79
Symptom	90.6 ± 12.8	92.5 ± 9.7	89.0 ± 16.7	92.1 ± 11.9	0.14
Daily Activities	92.7 ± 11.7	91.2 ± 12.8	90.1 ± 14.7	90.3 ± 14.8	0.441
Treatment concern	88.5 ± 12.4	88.7 ± 12.0	89.2 ± 11.7	88.5 ± 11.9	0.972
Satisfaction	84.9 ± 19.4	85.2 ± 18.9	84.4 ± 19.3	87.1 ± 16.0	0.684
Absolute changes in AFEQT score during 1 year					
Overall	19.7 ± 18.2	16.2 ± 17.3	11.9 ± 15.8	14.9 ± 17.4	<0.005
Symptom	20.3 ± 22.7	20.6 ± 21.0	12.4 ± 22.4	15.5 ± 20.5	<0.01
Daily Activities	17.7 ± 23.4	13.9 ± 23.0	10.5 ± 17.8	14.1 ± 21.7	0.08
Treatment concern	22.1 ± 18.6	16.3 ± 17.1	13.7 ± 16.1	15.4 ± 18.3	<0.005
Satisfaction	24.4 ± 25.9	20.2 ± 26.7	21.9 ± 24.5	27.0 ± 22.4	0.249

All data are expressed as the mean ± standard deviation.

**Table 3:** The comparison of changes in AFEQT score between normal and abnormal BNP groups adjusted by clinically relevant factors.

	Normal BNP *n* = 121	Abnormal BNP *n* = 370	*p* Value
Overall	18.2 ± 1.2 15.9–20.6	13.8 ± 0.7 12.5–15.1	<0.005
Symptom	20.2 ± 1.6 17.1–23.3	15.2 ± 0.8 13.5–16.8	<0.005
Daily Activities	15.8 ± 1.6 12.7–18.8	12.4 ± 0.9 10.7–14.1	0.055
Treatment concern	20.8 ± 1.3 18.3–23.3	14.8 ± 0.7 13.5–16.1	<0.001
Satisfaction	24.3 ± 1.8 20.8–27.8	22.5 ± 1.0 20.6–24.5	0.385

All data are expressed as the mean ± standard error and 95% confidence interval.

## Data Availability

KiCS AF Registry data can be found at https://center6.umin.ac.jp/cgi-open-bin/ctr/ctr_view.cgi?recptno=R000025601 (accessed on 29 September 2020).

## Supplementary Materials

The following supporting information can be downloaded at https://www.mdpi.com/article/10.3390/jcm13020407/s1, Figure S1: Impact of plasma brain natriuretic peptide levels on changes in the AFEQT score of each domain after catheter ablation in patients with atrial fibrillation; Table S1: The changes of AFEQT score adjusted by baseline AFEQT score; Table S2: The changes of AFEQT score adjusted by clinically relevant factors.
