# Supplementary material for: Clinical Utility of Baseline Brain Natriuretic Peptide Levels on Health Status Outcomes after Catheter Ablation for Atrial Fibrillation in Individuals without Heart Failure
Source: J Clin Med. 2024 Jan 11;13(2):407. doi: 10.3390/jcm13020407 (PMC10816027; doi:10.3390/jcm13020407)
Supplement: Supplementary file 1 [file jcm-13-00407-s001.zip › jcm-2805645-supplementary.pdf]

Supplemental Material

(A)

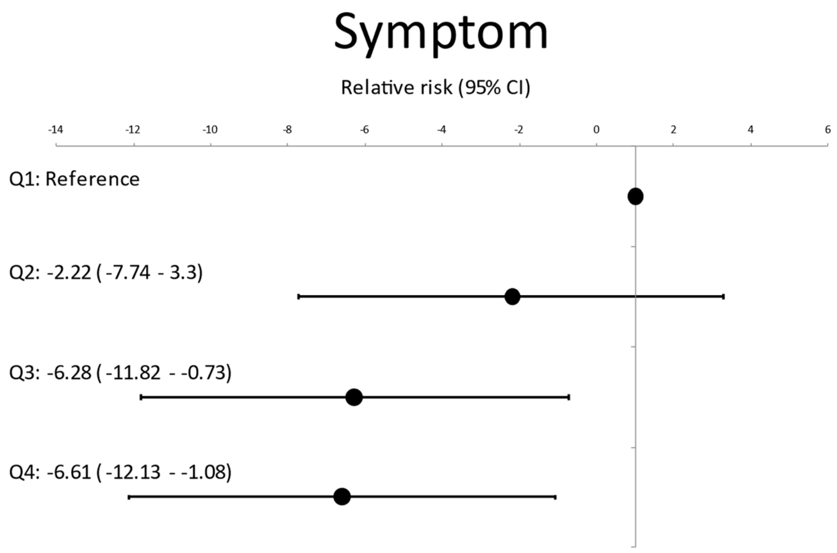

(B)

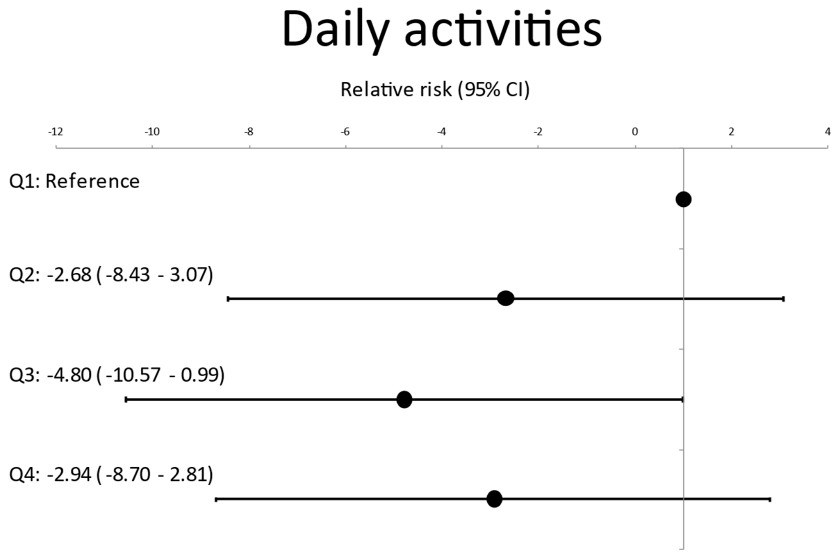

(C)

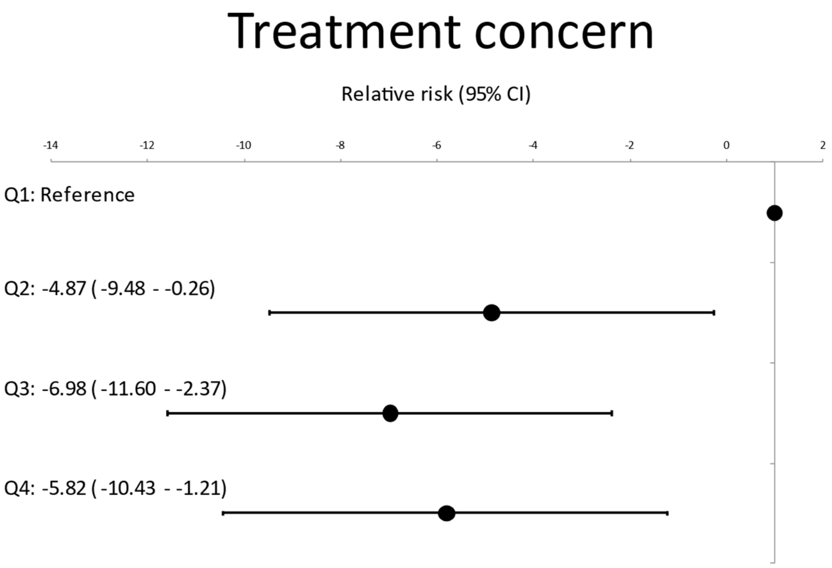

(D)

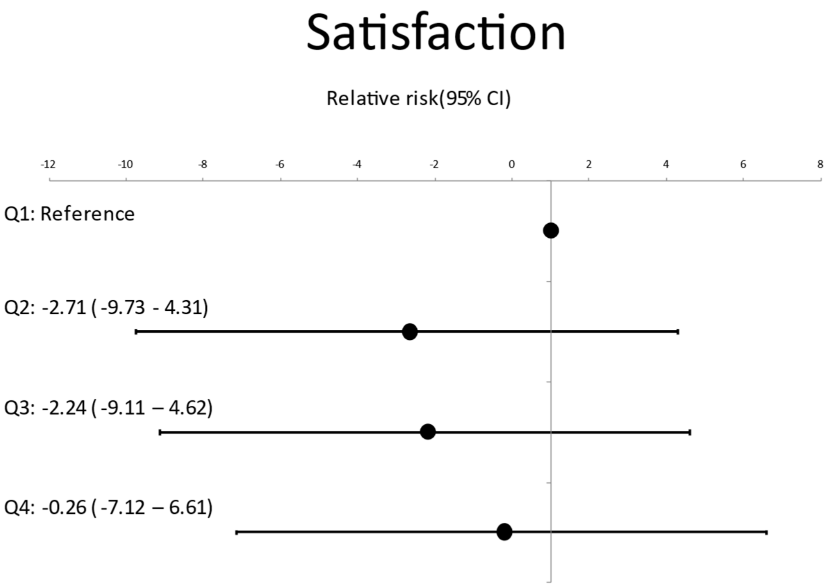

Supplemental Figure S1. Impact of plasma brain natriuretic peptide levels on changes in the AFEQT score of each domain after catheter ablation in patients with atrial fibrillation. (A) Symptom, (B) Daily activities, (C) Treatment concern, and (D) Satisfaction. Q1: 1.32–18.5 pg/mL, Q2: 18.7–37.7 pg/mL, Q3: 37.9–63.5 pg/mL, and Q4: 63.8–99.8 pg/mL.

Supplemental Table S1. The changes of AFEQT score adjusted by baseline AFEQT score

	Quartile 1 (Lowest, 1.32-18.5) n=123	Quartile 2 (18.7-37.7) n=123	Quartile 3 (37.9-63.5) n=122	Quartile 4 (Highest, 63.8-99.8) n=123	p value
Overall	16.9 ± 1.2<br>14.6-19.3	14.4 ± 1.1<br>12.2-16.6	12.2 ± 1.1<br>10.0-14.5	13.9 ± 1.2<br>11.5-16.3	<0.05
Symptom	19.3 ± 1.6<br>16.2-22.4	17.5 ± 1.5<br>14.5-20.6	13.7 ± 1.5<br>10.8-16.6	13.7 ± 1.4<br>11.0-16.4	<0.05
Daily Activities	14.1 ± 1.5<br>11.1-17.0	12.3 ± 1.4<br>9.5-15.1	10.6 ± 1.4<br>7.8-13.4	13.2 ± 1.6<br>10.0-16.3	0.403
Treatment concern	20.0 ± 1.3<br>17.5-22.5	15.4 ± 1.1<br>13.2-17.7	13.4 ± 1.2<br>11.1-15.8	15.1 ± 1.2<br>12.8-17.4	<0.001
Satisfaction	24.6 ± 1.7<br>21.1-28.0	21.7 ± 1.8<br>18.2-25.2	22.1 ± 1.6<br>19.1-25.3	24.2 ± 1.9<br>20.5-28.0	0.559

All data are sexpressed as the mean ± standard error and 95% confidence interval.

Supplemental Table S2. The changes of AFEQT score adjusted by clinically relevant factors.

	Quartile 1 (Lowest, 1.32-18.5) n=123	Quartile 2 (18.7-37.7) n=123	Quartile 3 (37.9-63.5) n=122	Quartile 4 (Highest, 63.8-99.8) n=123	p value
Overall	18.2 ± 1.2<br>15.9-20.6	15.0 ± 1.1<br>12.9-17.2	12.6 ± 1.2<br>10.3-14.9	13.6 ± 1.2<br>11.3-16.0	<0.005
Symptom	20.2 ± 1.6<br>17.1-23.3	18.0 ± 1.5<br>15.0-21.0	13.9 ± 1.5<br>11.0-16.8	13.6 ± 1.4<br>10.9-16.3	<0.005
Daily Activities	15.8 ± 1.5<br>12.7-18.9	13.1 ± 1.4<br>10.3-16.0	11.0 ± 1.4<br>8.2-13.9	12.9 ± 1.6<br>9.7-16.1	0.169
Treatment concern	20.7 ± 1.2<br>18.2-23.2	15.8 ± 1.1<br>13.6-18.1	13.7 ± 1.2<br>11.3-16.1	14.9 ± 1.2<br>12.6-17.2	<0.001
Satisfaction	24.3 ± 1.8<br>20.8-27.7	21.5 ± 1.8<br>18.0-25.1	22.0 ± 1.6<br>18.8-25.2	24.0 ± 1.9<br>20.2-27.7	0.613

All data are expressed as the mean ± standard error and 95% confidence interval.
